# Safety of Mechanical Thrombectomy for Acute Ischemic Stroke in Patients with Thrombocytopenia

**DOI:** 10.2174/0115672026296867240626120014

**Published:** 2024-07-10

**Authors:** Sujie Zheng, Fang Liu, Liang Yu, Xinzhao Jiang, Xiaoyan Wen, Xu Wang, Zongjie Shi

**Affiliations:** 1 Department of Clinical Laboratory, Laboratory Medicine Center, Zhejiang Provincial People's Hospital (Affiliated People's Hospital, Hangzhou Medical College), Hangzhou, Zhejiang, 310014, China;; 2 Department of Neurology, Center for Rehabilitation Medicine, Zhejiang Provincial People's Hospital (Affiliated People's Hospital, Hangzhou Medical College), Hangzhou, Zhejiang, 310014, China;; 3 Department of Radiology, Cancer Center, Zhejiang Provincial People's Hospital (Affiliated People's Hospital, Hangzhou Medical College), Hangzhou, Zhejiang, 310014, China;; 4 Department of Radiology, Center for Rehabilitation Medicine, Zhejiang Provincial People's Hospital (Affiliated People's Hospital, Hangzhou Medical College) Hangzhou, 310014, China

**Keywords:** Acute ischemic stroke, mechanical thrombectomy, neurointervention, sICH, large-vessel occlusion stroke, atrial fibrillation

## Abstract

**Background and Aim:**

The impact of low platelet count on outcomes in patients with Acute Ischemic Stroke (AIS) undergoing Mechanical Thrombectomy (MT) is still unclear. In this study we have further explored the effect of thrombocytopenia on the safety and efficacy of MT in patients with anterior circulation Large Vessel Occlusion (LVO) stroke.

**Materials and Methods:**

Patients with AIS who underwent MT at our center between June 2015 and November 2021 were examined. Based on the platelet count recorded on admission patients were divided into two groups: those with thrombocytopenia (<150 × 10^9^/L) and those without thrombocytopenia (≥ 150 × 10^9^/L). Symptomatic Intracranial Hemorrhage (sICH) was the primary safety outcome. The efficacy outcome was functional independence defined as a 90-day modified Rankin Scale (mRS) score of 0-2. Multivariate logistic regression models were used to determine the risk factors for post-procedure sICH and 90-day functional outcomes.

**Results:**

Among 302 patients included in the study, thrombocytopenia was detected in 111 (36.8%) cases. Univariate analysis showed age, the proportion of atrial fibrillation, the rates of sICH, 90-day poor outcomes, and mortality to be higher in patients with thrombocytopenia (all *p* <0.05). Multivariable analysis showed thrombocytopenia to be independently associated with a higher rate of sICH (OR 2.022, 95% CI 1.074-3.807, *p* =0.029) however, thrombocytopenia did not affect the 90-day functional outcomes (OR 1.045, 95% CI 0.490-2.230, *p* = 0.909) and mortality (OR 1.389, 95% CI 0.467– 4.130 *p* = 0.554).

**Conclusion:**

Thrombocytopenia may increase the risk of sICH but not affect the 90-day functional outcomes and mortality in patients with AIS treated with MT.

## 1 INTRODUCTION

Mechanical Thrombectomy (MT) is a standard minimally invasive procedure used to treat patients with Acute Ischemic Stroke (AIS) caused by anterior circulation Large Vessel Occlusion (LVO) [1]. Although in most cases, recanalization is timely and successfully achieved, many patients still have a poor prognosis [2]. The major limitations of MT include technical difficulty in navigating the wire through delicate intracranial vessels, limited access to trained neuro-interventionalists,   trauma   to   vessels,   and vasospasm. In addition, symptomatic intracranial hemorrhage is one of the most serious complications after MT.

Over the years, some studies have suggested that MT combined with recombinant tissue Plasminogen Activator (rt-PA) can improve the outcomes in patients with acute stroke caused by LVO. However, the latest Guidelines for the Early Management of Acute Ischemic Stroke do not recommend using IV-tPA in patients with a platelet count below <100,000/mL [1]. Yang *et al.* found thrombocytopenia to be associated with a higher in-hospital mortality, a longer length of stay, a higher rate of intracranial hemorrhage, a higher proportion of post-thrombolytic bleeding, and mechanical ventilation in patients who received intravenous thrombolysis [3]. Furthermore, thrombocytopenia has been recognized as an independent predictor of adverse clinical outcomes and associated with higher mortality in patients with AIS [4, 5]. However, the effect of thrombocytopenia on the safety and efficacy of MT is not clear. Recently, few studies have attempted to investigate this problem. Ždraljević *et al.* found thrombocytopenia to increase the mortality of patients with MT, but it did not increase the rate of symptomatic intracranial hemorrhage and had no significant effect on 90-day clinical outcomes [6]. In this study, we have further explored the effect of thrombocytopenia on the safety and efficacy of mechanical thrombectomy in patients with anterior circulation LVO stroke.

## 2 MATERIALS AND METHODS

### 2.1 Study Population

We retrospectively recruited patients with AIS [caused by anterior circulation Large Vessel Occlusion (LVO)] who have undergone MT with or without IV-tPA at Zhejiang Provincial People's Hospital (Affiliated People's Hospital, Hangzhou Medical College) between June 2015 and November 2021. Inclusion criteria were (1)LVO confirmed by CT Angiography (CTA) within 6 h of symptom onset, with LVO including the internal carotid artery and the middle cerebral artery (M1 or M2), and beyond 6 h of symptom onset according to the DEFUSE-3 and DAWN trial criteria [7, 8]; (2) patients who have achieved successful reperfusion after the procedure.

### 2.2 Baseline Data Collection

Baseline demographic, clinical, laboratory, and radiographic data were collected for analysis, including age, sex, comorbid conditions (history of smoking, hypertension, diabetes mellitus, and atrial fibrillation), baseline National Institute of Health Stroke Scale (NIHSS) [9], Alberta Stroke Program Early Computed Tomography Score (ASPECTS) [10], time of onset, blood pressure and glycemia on admission, etiology, occlusion site, and information on MT and procedural time. Based on the platelet count recorded on admission, patients were divided into two groups: those with thrombocytopenia (<150 × 10^9^/L) and those without thrombocytopenia (≥ 150 × 10^9^/L).

### 2.3 Outcome Analysis

The degree of reperfusion after the MT procedure was assessed by a modified Thrombolysis in Cerebral Infarction (mTICI) score [11]; an mTICI score of 2b or 3 indicated successful reperfusion. The primary safety outcome was symptomatic Intracranial Hemorrhage (sICH), which was analyzed according to the ECASS-III criteria [12]; sICH was defined as hemorrhage with neurologic deterioration of NIHSS score 4 or any hemorrhage leading to death. The efficacy outcome was 90-day functional independence, defined as a modified Rankin Scale (mRS) score of 0-2.

### 2.4 Statistical Analysis

Continuous variables have been presented as mean ± Standard Deviation (SD) or median with Interquartile Range (IQR), and categorical variables have been reported as numbers and percentages. Groups were compared using the student’s t-test and Mann-Whitney U-test for continuous variables or Chi-square test or Fisher’s exact test for categorical variables. Univariable analysis and multivariable logistic regression analysis were also performed. In order to adjust for known confounders and identify independent predictors of the 90-day functional outcomes and sICH, clinical variables with *p* < 0.05 from univariate analyses were included in multivariable logistic regression. *p* < 0.05 was considered statistically significant. Statistical analysis was performed using IBM SPSS statistics 23 (IBM, Armonk, New York, USA).

## 3 RESULTS

Overall, 302 patients were included in the study. The mean age was 71 years, and 59.9% of the patients were male. The median platelet count was 167× 10^9^/L (130-201× 10^9^/L); 36.8% (111/302) cases with a platelet count <150 × 10^9^/L had thrombocytopenia, and 7.6% (23/302) of these patients had a platelet count <100 × 10^9^/L on admission.

Baseline demographic data and clinical, laboratory, and radiographic features on admission are presented in Table **1**. In the univariate analysis, there was no significant difference in sex, comorbid conditions (history of smoking, hypertension, diabetes mellitus), baseline NIHSS, baseline SBP, procedure time, location of occlusion site, treatment with intravenous thrombolysis, or type of anesthesia between patients with thrombocytopenia and those without thrombocytopenia (all *p* >0.05). A group with thrombocytopenia was older [75 (66-82) *vs*. 69 (57-78), *p* =0.00^1^], while the percentage of atrial fibrillation was more common in the group with thrombocytopenia (72.1% *vs*. 50.3%, *p* =0.001). In addition, there was a statistically significant difference in the location of the occlusion site and stroke etiology between these two groups; also, the percentage of middle cerebral artery occlusion and cardiogenic embolism were higher in the group with thrombocytopenia (all *p* <0.05).

In the univariate analysis, the proportion of sICH was higher (26.1% *vs*. 15.7%, *p* =0.028), the proportion of good outcomes was lower (39.6% *vs*. 53.9%, *p* =0.017), and the proportion of mortality was higher in those with thrombocytopenia (19.8% *vs*. 10.5%, *p* =0.024) (Table **1**).

After controlling for hypertension, baseline ASPECTS, and glucose level on admission, multivariate regression analysis confirmed thrombocytopenia to increase the risk of sICH (OR 2.022, 95% CI 1.074-3.807; *p* = 0.029) (Table **2**). However, multivariate analysis showed that thrombocytopenia did not affect the 90-day functional outcomes (OR 1.045, 95% CI 0.490-2.230; *p* = 0.909) after controlling for age, male, baseline NIHSS, baseline SBP, platelet count,glucose level on admission, location of occlusion site, and sICH (Table **3**). In addition, after controlling for age, baseline NIHSS, baseline DBP,platelet count, and glucose level on admission, multivariate regression analysis showed that thrombocytopenia did not affect the 90-day mortality (OR 1.389, 95% CI 0.467-4.130; *p* = 0.554) (Table **4**).

## 4 DISCUSSION

This study has explored the influence of thrombocytopenia on the safety and efficacy of MT in patients with anterior circulation large-vessel occlusive stroke. Our data have suggested thrombocytopenia to increase the risk of sICH in patients with LVO-associated AIS who have undergone MT. However, thrombocytopenia did not affect the 90-day functional outcomes and mortality of MT.

The incidence of thrombocytopenia (with a platelet count < 150 × 10^9^/L) in our study group was 36.8%, while 7.6% of these patients had a platelet count <100 × 10^9^/L at admission. Other similar studies have reported a lower incidence of thrombocytopenia (9.6%, 13.3%, and 15%) [6, 13, 14]. This difference may be attributed to the different races of our study population. In addition, the median platelet count in the whole cohort in the present study was lower than that reported in a previous study [6]. Yet, the etiology of thrombocytopenia in our study was not clear. Previous studies have suggested immune-mediated disease or severe kidney disease to be the most common cause of thrombocytopenia [6, 13].

In our study, the patients with thrombocytopenia were older than those without thrombocytopenia, consistent with a previous study [6]. The effect of age on platelet count was also found in healthy people, and the platelet count gradually decreased with age [15]. However, the mechanism of platelet reduction in old age remains unclear.

Furthermore, our results have shown atrial fibrillation to be significantly higher in patients with thrombocytopenia, being consistent with a previous study [16]. Therefore, the etiology of cardio-embolism in the thrombocytopenia group was higher. Moreover, baseline ASPECTS was lower in the thrombocytopenia group, indicating that thrombocytopenia may lead to a larger infarct volume. Tohgi *et al.* showed a significant positive correlation between platelet aggregation and infarct size in the acute phase of stroke, and suggested that increased platelet aggregation, which decreases platelet count, leads to larger infarcts [17].

Our study has shown that thrombocytopenia increases the risk of sICH, inconsistent with previous studies [6, 13, 14]. The explanation may be that the patients in our study had lower platelet counts; the median platelet count of patients with thrombocytopenia was 121 (107-136) × 10^9^/L, while it has been reported to be 131 (119-137) × 10^9^/L in other studies [6]. Moreover, in this study, the proportion of intravenous thrombolysis in patients with thrombocytopenia was 34.2%, while this proportion was 10.5%in another study [6]. Yang *et al.* confirmed thrombocytopenia to be associated with a higher risk of intracranial hemorrhage in patients with AIS who received intravenous thrombolysis, and the intracranial hemorrhage rate was 1.8-fold higher in patients with thrombocytopenia than in those without [3]. However, our results showed thrombocytopenia to not affect the 90-day functional outcomes, in line with previously published studies [6, 13, 14]. A recent meta-analysis compared outcomes in patients with low *versus* normal platelet count at admission following MT for LVO and showed no difference in postprocedural clinical outcomes between the two groups [18]. Different from these results, Mönch *et al.* [14] found a marked decline in platelet count (>26%) to be associated with poorer clinical outcomes. Based on the published data, we speculate that thrombocytopenia might not affect short-term functional outcomes in patients who have undergone MT. However, for patients with a marked decline in platelet count post-procedure, its impact on prognosis should be considered.

Previous studies have suggested thrombocytopenia to be associated with higher short-term mortality in patients with AIS caused by anterior circulation LVO who have undergone MT. This may likely be due to more comorbidities and higher rates of poor baseline functional status in these patients [13]. The most common cause of death in patients with thrombocytopenia was a malignant cerebral infarction [6]. Contrary, our results showed that thrombocytopenia did not affect short-term mortality. In our study, age and the proportion of atrial fibrillation were higher in the thrombocytopenia group, and there was no significant difference in comorbidities and baseline NIHSS score between the two groups. Accordingly, higher short-term mortality was not detected in the thrombocytopenia group in the current study.

This study has involved a few limitations. First, this was a single-center retrospective study. We adjusted the patient baseline characteristics using multivariate logistic regression analysis to rule out possible bias. Second, pre-existing medical comorbidities that may potentially result in abnormal blood platelet count and adversely affect the patients' functional outcomes were not fully documented in this study. Third, the proportion of thrombocytopenia in our study population was higher, but the causes of thrombocytopenia were not fully documented, which may have affected the follow-up analysis. However, as mentioned in previous studies, the etiology of most thrombocytopeniaoccurrences was unknown.

## CONCLUSION

Thrombocytopenia may increase the risk of sICH but not affect the 90-day functional outcomes and mortality in patients with AIS treated with MT.

## AUTHORS’ CONTRIBUTIONS

The study is designed by SZ and ZS. Data is collected by XW and XW. Whereas, the interpretation of the data is done by LY and XJ.

## Figures and Tables

**Table 1:** Baseline characteristics of the study population.

**Characteristics**	**Those with Thrombocytopenia (n=111)**	**Those without Thrombocytopenia (n=191)**	***p*-value**
Male, n (%)	62 (55.9)	119 (62.3)	0.270
Age (y), median (IQR)	75 (66-82)	69 (57-78)	0.001
Hypertension, n (%)	73 (65.8)	134 (70.2)	0.428
Diabetes mellitus, n (%)	14 (12.6)	34 (17.8)	0.234
Atrial fibrillation, n (%)	80 (72.1)	96 (50.3)	0.001
Smoking, n (%)	23 (20.7)	43 (22.5)	0.716
Baseline NIHSS, median (IQR)	19 (15-25)	18 (14-23)	0.061
Baseline ASPECTS, median (IQR)	8 (6-10)	9 (7-10)	0.023
Baseline SBP (mmHg), median (IQR)	151 (137-166)	150 (132-166)	0.354
Baseline DBP (mmHg), median (IQR)	86 (78-98)	86 (76-97)	0.604
TOAST classification, n (%)	-	-	0.019
LAA	20 (18)	53 (27.7)	-
CE	77 (69.4)	101 (52.9)	-
UE	14 (12.6)	37 (19.4)	-
Laboratory finding, median (IQR)	-	-	-
Platelet count (x109 /L)	121 (107-136)	191 (171-214)	0.001
Glucose level on admission (mmol/L)	6.68 (6.11-7.6)	6.85 (6.16-8.67)	0.112
Creatinine (µmol/L)	66.8 (57.4-81.9)	67.2 (55.3-76.2)	0.509
Procedure time (min), median (IQR)	-	-	
OPT	340 (248-460)	329 (253-475)	0.927
ORT	407 (300-537)	388 (309-557)	0.934
Location of occlusion site, n (%)	-	-	0.012
ICA	46 (30.7)	63 (40.4)	-
MCA-M1	93 (62)	91 (58.3)	-
MCA-M2	11 (7.3)	2 (1.3)	-
Intravenous thrombolysis, n (%)	38 (34.2)	83 (43.5)	0.115
General anesthesia, n (%)	88 (79.3)	146 (76.4)	0.569
sICH, n (%)	29 (26.1)	30 (15.7)	0.028
90d mRS 0-2	44 (39.6)	103 (53.9)	0.017
Mortality	22 (19.8)	20 (10.5)	0.024

**Abbreviations:** IQR, Interquartile range; NIHSS, National Institutes of Health Stroke Scale; ASPECTS, Alberta Stroke Program Early CT Score; SBP, Systolic blood pressure; DBP, Diastolic blood pressure; TOAST, Trial of Org 10172 in Acute Stroke Treatment; LAA, Large artery atherosclerosis; CE, Cardioembolism; UE, Undetermined etiology; OPT, Onset to puncture time; ORT, Onset to reperfusion time; ICA, Internal carotid artery; MCA-M1, M1 segment of the middle cerebral artery; MCA-M2, M2 segment of the middle cerebral artery; sICH, symptomatic intracranial hemorrhage.

**Table 2:** Univariate and multivariate logistic regression analyses of risk factors for sICH in AIS patients with MT.

**-**	**OR (95%CI)**			
	**Unadjusted**	***p*-value**	**Adjusted**	***p*-value**
Hypertension	0.471 (0.265-0.838)	0.010	0.434(0.229-0.819)	0.010
Baseline ASPECTS	0.787 (0.698-0.886)	0.001	0.802(0.709-0.908)	0.001
TP	1.889 (1.071-3.331)	0.028	2.022(1.074-3.807)	**0.029**
Glucose level on admission	1.136 (1.021-1.263)	0.019	1.201(1.064-1.356)	0.003

**Abbreviations:** ASPECTS, Alberta Stroke Program Early CT Score; TP, Thrombocytopenia.

**Table 3:** Univariate and multivariate logistic regression analyses of risk factors for 90-day poor outcomes in AIS patients with MT.

**-**	**OR (95%CI)**			
**-**	**Unadjusted**	***p*-value**	**Adjusted**	***p*-value**
Age	1.046 (1.027-1.066)	0.001	1.034 (1.012-1.058)	0.003
Male	0.568 (0.358-0.903)	0.017	0.913 (0.525-1.590)	0.913
Baseline NIHSS	1.119 (1.074-1.165)	0.001	1.105 (1.056-1.156)	0.001
Baseline SBP	1.010 (1.001-1.020)	0.030	1.007 (0.997-1.018)	0.177
Platelet count	0.994 (0.990-0.999)	0.010	0.997 (0.991-1.004)	0.418
TP	1.702 (1.064-2.723)	0.027	1.045 (0.490-2.230)	0.909
Glucose level on admission	1.151 (1.035-1.280)	0.009	1.176 (1.048-1.320)	0.006
Location of the occlusion site	0.586 (0.386-0.892)	0.013	0.572 (0.351-0.932)	0.025
sICH	4.684 (2.414-9.086)	0.001	3.279 (1.561-6.889)	0.002

**Abbreviations:** NIHSS, National Institutes of Health Stroke Scale; SBP, Systolic blood pressure; TP, Thrombocytopenia; sICH, symptomatic intracranial hemorrhage.

**Table 4:** Univariate and multivariate logistic regression analyses of risk factors for 90-day mortality in AIS patients with MT.

**-**	**OR (95%CI)**			
**-**	**Unadjusted**	***p*-value**	**Adjusted**	***p*-value**
Age	1.042 (1.012-1.073)	0.006	1.038 (1.005-1.071)	0.024
Baseline NIHSS	1.060 (1.008-1.115)	0.023	1.052 (0.997-1.111)	0.067
Baseline DBP	0.978 (0.957-0.999)	0.038	0.975 (0.954-0.998)	0.029
Platelet count	0.993 (0.986-0.999)	0.028	0.996 (0.985-1.007)	0.442
TP	2.113 (1.095-4.079)	0.026	1.389 (0.467-4.130)	0.554
Glucose level on admission	1.156 (1.030-1.297)	0.014	1.216 (1.067-1.387)	0.003

**Abbreviations:** NIHSS, National Institutes of Health Stroke Scale; DBP, Diastolic blood pressure; TP, Thrombocytopenia.

## Data Availability

The data and supportive information are available within the article.

## LIST OF ABBREVIATIONS

AIS: Acute Ischemic Stroke
ASPECTS: Alberta Stroke Program Early Computed Tomography Score
LVO: Large Vessel Occlusion
MT: Mechanical Thrombectomy
NIHSS: National Institute of Health Stroke Scale

## STANDARD OF REPORTING

STROBE guideline were followed.
